# Associations of overweight and obesity with cardiometabolic risk factor clusters among Korean adolescents

**DOI:** 10.1038/s41598-024-53698-6

**Published:** 2024-02-13

**Authors:** Youn Huh, Hye Soon Park

**Affiliations:** 1 Department of Family Medicine, Uijeongbu Eulji Medical Center, Eulji University, Gyeonngi-do, Republic of Korea; 2 Department of Family Medicine, Asan Medical Center, University of Ulsan College of Medicine, 88 Olympic-ro 43-gil, Songpa-gu, Seoul, 05505 Republic of Korea

**Keywords:** Health care, Risk factors

## Abstract

This study investigated the association between overweight, obesity, and cardiometabolic risk factor clusters in Korean adolescents. We included 2182 participants (1161 boys and 1021 girls) aged 12–18 years from the Korea National Health and Nutrition Examination Survey (2016–2021). Cardiometabolic risk factors include hypertension, high low-density lipoprotein cholesterol (LDL-C) level, low high-density lipoprotein cholesterol (HDL-C) level, hypertriglyceridemia, high fasting plasma glucose level, elevated alanine aminotransferase level, and hyperuricemia. The average age was 15.1 ± 0.1 years in both boys and girls. The proportion of subjects with ≥ 1, ≥ 2, and ≥ 3 cardiometabolic risk factors was 76.5%, 49.8%, and 22.7% in obese adolescents, and 60.5%, 24.0%, and 9.1%, in overweight adolescents, respectively. Compared to adolescents with underweight/normal weight, the odds ratios (ORs) and 95% confidence intervals for the clustering of cardiometabolic risk factors were at 2.76 (1.74–4.38) for ≥ 1; 3.75 (2.11–6.66) for ≥ 2; and 4.75 (1.70–13.25) for ≥ 3 factors in obese adolescents and 1.88 (1.26–2.81) for ≥ 1 factor in overweight adolescents. Overweight and obese adolescents exhibited high cardiometabolic risk clustering. Prevention and management of overweight/obesity in Korean adolescents are emerging to mitigate health risk associated with this condition.

## Introduction

The worldwide prevalence of overweight and obese adolescents and children increased 4.5 fold between 1975 and 2016^1^. Among American adolescents aged 12–19 years, the prevalence of obesity has risen steadily, while this trend is more pronounced in boys than in girls^2,3^. A climbing prevalence of adolescent obesity has also been reported in South Korea. Rates of overweight/obesity increased from 18.8% (boys: 17.3%; girls: 20.6%) in 2011 to 23.7% (boys: 24.0%; girls: 23.5%) in 2019^4^. Moreover, obese adolescents have an increased risk of obesity-related comorbidities such as cardiometabolic syndrome^5^, obstructive sleep apnea^6^, and non-alcoholic fatty liver disease (NAFLD)^7^. Additionally, medical service utilization and expenditure in children with obesity are more significant than those in children with normal weight, owing to the evaluation and management of comorbidities^8^.

Furthermore, obesity during childhood and adolescence is likely to persist into adulthood. Children who are overweight between 2 and 5 years of age are four times more likely to be obese in adulthood than children of normal weight were^9^. A previous study demonstrated that an increase in body mass index (BMI) among adolescents augments the risk of fatal and nonfatal cardiovascular disease (CVD) in both men and women, as well as mortality in adulthood^10^. In particular, severe obesity is associated with an immediate risk of CVD complications, including elevated blood pressure, blood glucose and lipid levels^11^. Adolescents with NAFLD confer a higher risk of developing fibrosis as adults^12^. Additionally, comorbidities related to overweight/obesity in adolescents lead to a poor quality of life and a large burden on the healthcare system^13^.

Overweight/obese adolescents present a greater tendency to persist into adulthood, compared overweight/obese children^14^. A previous study showed that adolescents with a high BMI had a 7–10 times risk of overweight/obesity in adulthood than those with a low BMI^14^. Additionally, the risk of CVD in adulthood increased with age in both sexes^10^. The Bogalusa Heart Study showed that adolescents with obesity have an increased risk of developing clustered cardiometabolic risk factors^15^. Furthermore, prevalence of metabolic syndrome, which is a cluster indicator of cardiometabolic risk, in overweight and obese adolescents was 30.3% in the United States^16^ and 40.4% in Germany^17^. A recent study in Korea showed that the prevalence of metabolically unhealthy adolescents among overweight/obese Korean youths in 2019 was 64.3%^4^. However, most studies on the clustering of cardiometabolic risk factors and their association with overweight/obesity were conducted in Western adolescents, and studies on Asian counterparts are limited. Therefore, this study aimed to investigate the association between overweight/obesity and clustering of cardiometabolic risk factors in Korean adolescents, including healthy and unhealthy individuals, using nationally representative data.

## Methods

### Data source and study participants

Data from the Korea National Health and Nutrition Examination Survey (KNHANES) between 2016 and 2020 were collected. The KNHANES includes a stratified, multistage probability sampling of household units that participated in the survey. It provides data on demographic characteristics, health behaviors, and health status obtained from personal interviews as well as data obtained from physical examinations performed in mobile examination centers, including anthropometric measurements and blood samples. The initial study sample initially included 3112 KNHANES participants aged 12–18 years between 2016 and 2021. We excluded 930 adolescents with missing data for any of the variables. The resultant study sample comprised 2182 adolescents (1021 boys and 1161 girls). The informed consent was obtained from all participants before the investigation begins and the KNHANES was approved by the Institutional Review Board of the Korea Centers for Disease Control and Prevention (IRB No: 2018-01-03-P-A, 2018-01-03-C-A, and 2018-01-03-2C-A). The present study was carried out in accordance with the Declaration of Helsinki.

### Definitions of obesity and cardiometabolic risk factors

The height of the participants was measured to the nearest 0.1 cm using a stadiometer (SECA 225; Hamburg, Germany). Body weight was measured to the nearest 0.1 kg by using a balance scale (GL-6000-20; Cas, Yangju, Korea). Waist circumference (WC) was measured at the midpoint between the bottom of the subcostal region and the top of the iliac crest using a fiberglass tape. BMI was calculated by dividing the weight in kilograms by the square of height in meters. Participants were classified as underweight/normal weight (BMI < 85th percentile) or overweight (85th percentile ≤ BMI < 95th percentile) or obese (BMI ≥ 95th percentile) according to age- and sex-specific BMI percentiles provided by the 2017 Korean National Growth Charts for children and adolescents^18^. Abdominal obesity was defined as waist circumference divided by height ≥ 0.48^19^.

The cardiometabolic risk factors include hypertension, high low-density lipoprotein cholesterol (LDL-C), low high-density lipoprotein cholesterol (HDL-C), hypertriglyceridemia, high fasting plasma glucose (FPG), elevated alanine aminotransferase (ALT), and hyperuricemia. Systolic and diastolic blood pressure (BP) was measured three times in a sitting position using a standardized method with an internationally certified BP monitor with various cuff sizes, based on arm circumference; the mean values of the second and third BP measurements were determined. Blood samples were collected in the morning after an overnight fast. Hypertension was defined as systolic BP > 95th percentile or diastolic BP > 95th percentile for age, sex, and height, based on the 2017 Korean National Growth Chart for Children and Adolescents^18^. Blood samples were collected in the morning after overnight fasting. To define abnormal lipid profiles, we used the cut-off values for LDL-C (≥ 130 mg/dL), HDL-C (< 40 mg/dL), and triglycerides (≥ 130 mg/dL)^20^. High FPG was defined at ≥ 100 mg/dL^21^ and elevated ALT at > 40 IU/L. Hyperuricemia was defined as ≥ 6.0 mg/dL in subjects aged 12 years and ≥ 7.5 mg/dL in subjects aged 13–18 years based on reference values from the Mayo Clinic Laboratories^22^.

### Lifestyle factors

Household income levels were divided into first and second quartile groups, and others. Smoking status and alcohol consumption were dichotomized according to a history of smoking or alcohol consumption. Physical activity was categorized into two groups according to whether strength exercise was performed ≥ 3 times/week. A parental family history was defined as adolescents whose parents had at least one of the following conditions: hypertension, diabetes mellitus, or dyslipidemia. Breakfast was divided into whether or not participants had breakfast > 2 times/week and eating out was classified into whether or not participants ate out ≥ 2 times/day. Stress was divided into two groups based on stress recognition.

### Statistical analyses

We combined data from the 2016 to 2020 KNHANES using its raw data analysis guidelines. Based on a complex sample design, we conducted all analyses by assigning dispersed stratification estimates, stratification variables, and weighted sample values. Continuous variables were analyzed using a general linear model and presented as means and standard errors. Categorical variables were presented as ratios and standard errors and analyzed using the chi-square test. Furthermore, to determine the association between overweight and obesity, as the independent variable and the clustering of cardiometabolic risk factors, as the dependent variable, we conducted multivariable logistic regression analysis and calculated the odds ratios (ORs) and 95% confidence intervals (CIs) adjusted for sex, age, abdominal obesity, income, alcohol consumption, smoking status, physical activity, family history, breakfast consumption, eating habits, and stress levels. Statistical significance was set at *p* < 0.05. All analyses were performed using the SPSS software (version 24.0; IBM Corp., Armonk, NY, USA).

### Ethical approval

This study was approved by the Institutional Review Board of the Korea Center for Disease Control and Prevention (IRB No: 2018-01-03-P-A, 2018-01-03-C-A, and 2018-01-03-2C-A) approved the KNHANES.

### Consent to participate

The requirement for informed consent was waived because the data were anonymized and de-identified.

## Results

### Basic characteristics of the study participants

Table 1 presents the basic characteristics of the adolescents included in this study. The mean age was 15.1 ± 0.1 years. The mean body weight and mean WC were 73.3 ± 0.5 kg and 83.4 ± 0.3 cm, respectively, in boys and 62.3 ± 0.4 kg and 75.6 ± 0.3 cm, respectively, in girls. The mean BMI value was 25.0 ± 0.1 kg/m^2^ in boys and 24.1 ± 0.1 kg/m^2^ in girls. The mean values of most cardiometabolic risk factors were worse in overweight and obese adolescents than in underweight and normal-weight adolescents. Frequency of positive parental family history was significantly higher in those with increased BMI. Economic status, smoking status, alcohol consumption, eating habits, and stress levels did not differ significantly between individuals with different obesity status.

**Table 1:** Baseline characteristics of the study participants.

	Total (n = 2182)			
	Underweight/normal	Overweight	Obesity	*p*-value
	(n = 1664)	(n = 199)	(n = 319)	
Age (years)	15.1 ± 0.1	15.0 ± 0.2	15.1 ± 0.1	0.792
Height (cm)	165.2 ± 0.3	166.8 ± 0.7	166.8 ± 0.5	0.003
Weight (kg)	54.4 ± 0.3	69.5 ± 0.8	81.4 ± 0.9	< 0.001
WC (cm)	68.3 ± 0.2	80.6 ± 0.5	90.9 ± 0.7	< 0.001
BMI (kg/m^2^)	19.8 ± 0.1	24.8 ± 0.1	29.1 ± 0.2	< 0.001
Systolic BP (mmHg)	107.6 ± 0.3	111.3 ± 0.9	115.8 ± 0.7	< 0.001
Diastolic BP (mmHg)	67.3 ± 0.3	69.6 ± 0.7	70.0 ± 0.6	< 0.001
TC (mg/dL)	160.7 ± 0.8	168.0 ± 2.4	171.3 ± 1.8	< 0.001
LDL-C (mg/dL)	91.9 ± 0.7	100.4 ± 2.1	103.7 ± 1.6	< 0.001
HDL-C (mg/dL)	52.7 ± 0.3	48.7 ± 0.6	44.3 ± 0.6	< 0.001
TG (mg/dL)	80.5 ± 1.3	94.9 ± 3.9	116.5 ± 3.7	< 0.001
FPG (mg/dL)	90.9 ± 0.2	92.3 ± 0.9	93.8 ± 0.6	0.002
AST (IU/L)	19.0 ± 0.2	20.3 ± 0.7	24.2 ± 0.9	< 0.001
ALT (IU/L)	13.7 ± 0.4	21.4 ± 2.3	33.0 ± 2.0	< 0.001
Uric acid (mg/dL)	5.3 ± 0.0	6.0 ± 0.1	6.4 ± 0.1	< 0.001
Income (low)	34.9 (1.6)	32.2 (3.7)	37.8 (3.5)	0.518
Current smoker	9.4 (0.9)	9.1 (2.4)	7.4 (1.6)	0.603
Drinker	28.0 (1.4)	23.5 (3.4)	27.2 (3.1)	0.533
Regular physical activity	18.4 (1.1)	14.5 (2.8)	15.1 (2.2)	0.264
Family history (+)	26.5 (1.3)	27.1 (3.7)	39.8 (3.3)	< 0.001
Breakfast (+)	69.8 (1.3)	73.1 (3.5)	68.4 (3.1)	0.613
Eating out (+)	13.8 (1.1)	7.6 (2.7)	13.0 (2.3)	0.188
Stress (+)	24.7 (1.2)	25.3 (3.3)	30.2 (2.6)	0.137

*ALT* alanine aminotransferase, *AST* aspartate aminotransferase, *BMI* body mass index, *BP* blood pressure, *FPG* fasting plasma glucose, *HDL-C* high-density lipoprotein cholesterol, *LDL-C* low-density lipoprotein cholesterol, *TC* total cholesterol, *TG* Triglyceride, *WC* Waist circumference.

Values are presented as mean value ± standard error (SE) or percentages (%).

### Prevalence of individual and clustered cardiometabolic risk factors according to obesity status

Tables 2 and 3 show the prevalence of individual and clustered cardiometabolic risk factors according to the obesity status of adolescents. The proportion of obese adolescents with hypertriglyceridemia was the highest (31.0%), followed by those with low HDL-C (29.9%) and hyperuricemia (27.2%). Overweight adolescents with hyperuricemia represented 23.0%, followed by those with hypertriglyceridemia (19.8%) and high LDL (14.4%). In boys, the proportion of obese participants with hyperuricemia was the highest (42.5%), followed by those with low HDL-C (36.7%), hypertriglyceridemia (29.2%), and hypertension (23.5%). Among girls, the proportion of obese subjects with hypertriglyceridemia was the highest (33.5%), followed by those with low HDL-C (20.6%), high FPG (19.6%), and hypertension (15.9%). Excluding high FPG levels in boys and high LDL-C levels in girls, the proportion of all cardiovascular risk factors was higher in both overweight and obese boys and girls than in their underweight/normal-weight counterparts.

**Table 2:** Association of individual and clustered cardiometabolic risk factors with overweight/obesity in Korean adolescents.

	Total (n = 2182)										
	Underweight/normal (n = 1664)		Overweight (n = 199)		Obesity (n = 319)		*p*-value	Underweight/normal	Overweight	Obesity	*p* for trend
	N	% (SE)	N	% (SE)	N	% (SE)		OR (95%CI)	OR (95%CI)	OR (95%CI)	
Cardiometabolic risk factors											
Hypertension	92	5.3 (0.7)	25	12.8 (2.9)	65	20.3 (2.6)	< 0.001	1 (ref.)	1.52 (0.75–3.09)	1.89 (0.97–3.70)	0.062
High LDL-C	88	5.2 (0.6)	26	14.4 (3.1)	36	11.3 (2.3)	< 0.001	1 (ref.)	**2.45 (1.26–4.77)**	1.39 (0.65–2.97)	0.368
Low HDL-C	124	7.4 (0.7)	22	11.6 (2.7)	95	29.9 (3.3)	< 0.001	1 (ref.)	1.23 (0.58–2.59)	**3.33 (1.70–6.52)**	< 0.001
Hypertriglyceridemia	177	10.1 (0.8)	39	19.8 (3.2)	104	31.0 (3.0)	< 0.001	1 (ref.)	1.28 (0.75–2.18)	1.62 (0.95–2.78)	0.079
High FPG	171	9.6 (0.8)	25	12.3 (2.6)	56	17.7 (2.4)	< 0.001	1 (ref.)	1.19 (0.67–2.11)	1.70 (0.94–3.07)	0.081
Elevated ALT	35	2.2 (0.4)	12	5.9 (1.6)	73	22.1 (2.7)	< 0.001	1 (ref.)	1.40 (0.62–3.14)	**4.42 (2.21–8.87)**	< 0.001
Hyperuricemia	132	7.9 (0.8)	43	23.0 (3.4)	96	27.2 (3.0)	< 0.001	1 (ref.)	**2.53 (1.30–4.95)**	**2.49 (1.24–4.99)**	0.015
Clustering cardiometabolic risk factors											
≥ 1	623	36.4 (1.4)	116	60.5 (4.1)	252	76.5 (2.8)	< 0.001	1 (ref.)	**1.88 (1.26–2.81)**	**2.76 (1.74–4.38)**	< 0.001
≥ 2	156	9.0 (0.8)	44	24.0 (3.7)	163	49.8 (3.4)	< 0.001	1 (ref.)	1.74 (0.97–3.11)	**3.75 (2.11–6.66)**	< 0.001
≥ 3	34	2.0 (0.4)	18	9.1 (2.2)	75	22.7 (2.6)	< 0.001	1 (ref.)	2.27 (0.78–6.57)	**4.75 (1.70–13.25)**	0.001

*ALT* alanine aminotransferase, *FPG* fasting plasma glucose, *HDL-C* high-density lipoprotein cholesterol, *LDL-C* low-density lipoprotein cholesterol.

*p*-values were presented as numbers and percentages (standard errors).

ORs and 95% confidence intervals (CIs) were obtained using multivariate logistic regression analysis after adjusting for sex, age, abdominal obesity, income, alcohol consumption, smoking status, physical activity, family history, breakfast consumption, eating habits, and stress level.

Significant values are in bold.

**Table 3:** Prevalence of individual and clustered cardiometabolic risk factors according to overweight/obesity status in Korean boys and girls.

	Boy (n = 1161)							Girl (n = 1021)						
	Underweight/normal (n = 861)		Overweight (n = 111)		Obesity (n = 189)		*p*-value	Underweight/normal (n = 803)		Overweight (n = 88)		Obesity (n = 130)		*p*-value
	N	% (SE)	N	% (SE)	N	% (SE)		N	% (SE)	N	% (SE)	N	% (SE)	
Cardiometabolic risk factors														
Hypertension	49	5.1 (0.8)	10	8.9 (3.0)	43	23.5 (3.6)	< 0.001	43	5.6 (1.0)	15	17.5 (4.2)	22	15.9 (3.7)	< 0.001
High LDL-C	29	3.3 (0.7)	15	16.7 (4.5)	21	9.7 (2.4)	< 0.001	59	7.2 (1.0)	11	11.7 (3.8)	15	13.6 (3.9)	0.082
Low HDL-C	87	10.3 (1.2)	16	16.7 (4.5)	65	36.7 (4.4)	< 0.001	37	4.4 (0.8)	6	5.4 (2.4)	30	20.6 (4.1)	< 0.001
Hypertriglyceridemia	80	8.5 (1.0)	26	24.7 (4.7)	61	29.2 (3.9)	< 0.001	97	11.8 (1.2)	13	13.8 (3.8)	43	33.5 (4.7)	< 0.001
High FPG	120	13.3 (1.4)	20	16.5 (3.7)	33	16.4 (2.8)	0.443	51	5.6 (0.9)	5	7.2 (3.2)	23	19.6 (3.7)	< 0.001
Elevated ALT	33	3.8 (0.8)	10	8.1 (2.6)	55	28.0 (3.7)	< 0.001	2	0.4 (0.3)	2	3.1 (1.4)	18	13.8 (3.4)	< 0.001
Hyperuricemia	124	14.3 (1.5)	40	37.4 (5.2)	85	42.5 (4.3)	< 0.001	8	1.0 (0.4)	3	5.6 (3.2)	11	6.0 (2.1)	0.001
Clustering cardiometabolic risk factors														
≥ 1	383	43.2 (2.0)	74	70.2 (4.8)	166	85.0 (3.1)	< 0.001	240	29.0 (1.9)	42	48.8 (6.0)	86	64.7 (4.8)	< 0.001
≥ 2	107	11.9 (1.3)	35	35.0 (5.4)	116	60.5 (4.2)	< 0.001	49	5.8 (0.9)	9	10.7 (3.1)	47	34.9 (4.9)	< 0.001
≥ 3	27	2.9 (0.6)	16	14.5 (3.7)	54	27.0 (3.5)	< 0.001	7	1.0 (0.4)	2	2.5 (1.8)	21	16.9 (3.8)	< 0.001

*ALT* alanine aminotransferase, *FPG* fasting plasma glucose, *HDL-C* high-density lipoprotein cholesterol, *LDL-C* low-density lipoprotein cholesterol.

Values were presented as numbers and percentages (standard errors).

**Figure 1:**
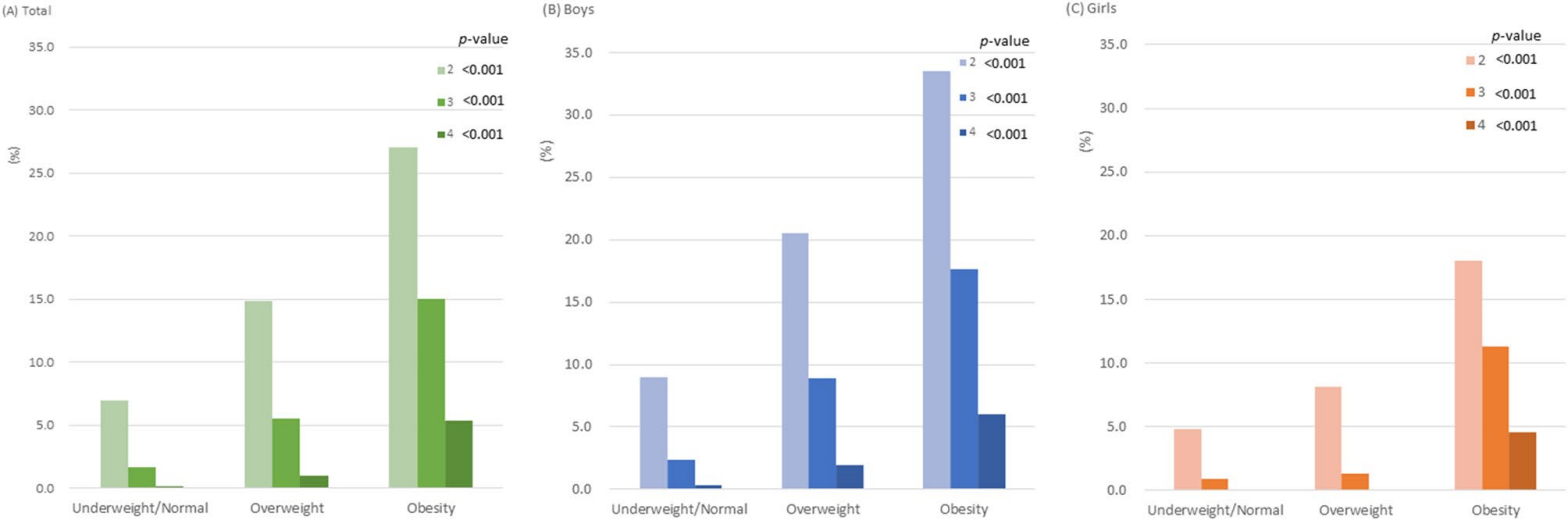
Prevalence of the number of clustering cardiometabolic risk factors according to overweight and obesity in adolescents.

Figure 1 shows the prevalence of the clustering of cardiometabolic risk factors according to obesity status in adolescents. The proportions of clusters with 2, 3, and 4 cardiometabolic risk factors were 27.0%, 15.0%, and 5.4%, in obese subjects; 14.9%, 5.5%, and 1.0%, in overweight subjects, and 7.0%, 1.7%, and 0.2%, in underweight/normal-weight subjects, respectively (all *p* < 0.001). The proportion of clusters with 2, 3, and 4 cardiometabolic risk factors in boys were 33.5%, 17.6%, and 6.0%, in the obese group and 20.5%, 8.9%, and 1.9%, in the overweight group, respectively (all *p* < 0.001). In girls, the prevalence of two and three clustered factors was 18.0% and 11.3% in the obesity group, and 8.1% and 1.3% in the overweight group (all *p* < 0.001).

The prevalence of overweight and obesity with at least one cardiometabolic risk factor was 60.5% and 76.5%, whereas that of their underweight/normal-weight counterparts was 36.4%. The proportion of boys who were overweight and obese and had at least one cardiometabolic risk factor was 70.2% and 85.0%, whereas that of their underweight/normal-weight counterparts was 43.2%. The proportion of girls who were overweight and obese and had at least one cardiometabolic risk factor was 48.8% and 64.7%, whereas that of their underweight/normal-weight counterparts was 29.0%. The proportion of subjects with ≥ 2 and ≥ 3 clustering cardiometabolic risk factors were 49.8% and 22.7%, in obese adolescents, and 24.0% and 9.1% in overweight adolescents, while this proportion was 9.0% and 2.0% in underweight/normal weight adolescents, respectively (all *p* < 0.001).

### ORs and 95% CIs of the clustering of cardiometabolic risk factors according to obesity status

Tables 2 and 4 present the multivariate analyses of the association between the clustering of cardiometabolic risk factors and overweight and obesity in adolescents. Obese adolescents had an increased OR for elevated ALT of 4.42 (95% CI 2.21–8.87) than adolescents with underweight/normal weight after adjusting for confounding variables. Compared with adolescents with underweight/normal weight, the ORs among obese adolescents were higher at 3.33 (1.70–6.52) for low HDL-C and 2.49 (1.24–4.99) for hyperuricemia and these among overweight adolescents were higher at 2.45 (1.26–4.77) for high LDL-C and 2.53 (1.30–4.95) for hyperuricemia. Compared with boys with underweight/normal weight, the ORs among obese adolescents were higher at 3.94 (1.75–8.87) for elevated ALT, 3.91 (1.81–8.45) for low HDL-C, and 2.55 (1.28–5.11) for hyperuricemia and these among overweight adolescents were higher at 3.93 (1.15–13.39) for high LDL-C and 2.49 (1.30–4.79) for hyperuricemia. In girls, obesity was associated with an increased OR for elevated ALT levels at 13.05 (2.84–59.96) compared to those with underweight/normal weight. Meanwhile, the OR among obese girls was 5.11 (1.90–13.68) for high FPG, and among overweight girls were 2.99 (1.43–6.25) for hypertension compared to their counterparts with underweight/normal weight.

**Table 4:** Adjusted ORs and 95% CIs for individual and clustered cardiometabolic risk factors according to overweight/obesity status in Korean boys and girls.

	Boys				Girls			
	Underweight/normal	Overweight	Obesity	*p* for trend	Underweight/normal	Overweight	Obesity	*p* for trend
Cardiometabolic risk factors								
Hypertension	1 (ref.)	0.66 (0.21–2.10)	1.42 (0.58–3.45)	0.268	1 (ref.)	**2.99 (1.43–6.25)**	1.97 (0.83–4.71)	0.059
High LDL-C	1 (ref.)	**3.93 (1.15–13.39)**	1.80 (0.46–7.06)	0.577	1 (ref.)	1.57 (0.68–3.60)	1.54 (0.65–3.66)	0.233
Low HDL-C	1 (ref.)	1.49 (0.60–3.71)	**3.91 (1.81–8.45)**	< 0.001	1 (ref.)	0.82 (0.25–2.75)	2.53 (0.71–9.01)	0.131
Hypertriglyceridemia	1 (ref.)	1.76 (0.84–3.68)	1.64 (0.82–3.25)	0.206	1 (ref.)	0.85 (0.38–1.90)	1.83 (0.74–4.50)	0.214
High FPG	1 (ref.)	1.08 (0.58–2.02)	0.92 (0.50–1.69)	0.787	1 (ref.)	1.35 (0.44–4.16)	**5.11 (1.90–13.68)**	0.002
Elevated ALT	1 (ref.)	1.22 (0.48–3.08)	**3.94 (1.75–8.87)**	< 0.001	1 (ref.)	4.24 (0.53–33.63)	**13.05 (2.84–59.96)**	< 0.001
Hyperuricemia	1 (ref.)	**2.49 (1.30–4.79)**	**2.55 (1.28–5.11)**	0.011	1 (ref.)	3.80 (0.44–33.06)	2.08 (0.22–19.81)	0.583
Clustering cardiometabolic risk factors								
≥ 1	1 (ref.)	**1.98 (1.11–3.55)**	**3.44 (1.76–6.71)**	< 0.001	1 (ref.)	**1.85 (1.07–3.17)**	**2.35 (1.23–4.49)**	0.005
≥ 2	1 (ref.)	2.04 (0.99–4.20)	**3.84 (1.95–7.56)**	< 0.001	1 (ref.)	1.25 (0.53–2.97)	**3.96 (1.45–10.78)**	0.006
≥ 3	1 (ref.)	2.97 (0.88–10.00)	**5.08 (1.73–14.91)**	0.001	1 (ref.)	1.07 (0.10–11.41)	4.98 (0.43–57.62)	0.121

*ALT* alanine aminotransferase, *CI* confidence interval, *FPG* fasting plasma glucose, *HDL-C* high-density lipoprotein cholesterol, *LDL-C* low-density lipoprotein cholesterol, *OR* odds ratio.

Values were obtained using multivariate logistic regression analysis after adjusting for age, abdominal obesity, income, alcohol consumption, smoking status, physical activity, family history, breakfast consumption, eating habits, and stress levels.

Significant values are in bold.

Compared to adolescents with underweight/normal weight, the ORs (95% CI) were higher at 2.76 (1.74–4.38); 3.75 (2.11–6.66); and 4.75 (1.70–13.25) in obese adolescents, for ≥ 1, ≥ 2, and ≥ 3 of clustering cardiometabolic risk factors respectively, and 1.88 (1.26–2.81) for ≥ 1 factor in overweight adolescents. Compared to adolescents with underweight/normal weight, the ORs (95% CI) for ≥ 1, ≥ 2, and ≥ 3 of clustering cardiometabolic risk factors were higher at 3.44 (1.76–6.71) for; 3.84 (1.95–7.56); and 5.08 (1.73–14.91) for ≥ 3 factors in obese boys and 2.35 (1.23–4.49) for ≥ 1; and 3.96 (1.45–10.78) for ≥ 2 factors in obese girls, respectively.

## Discussion

The present study confirmed that overweight and obese adolescents had an increased risk of clustering cardiometabolic risk factors compared with underweight/normal-weight adolescents of both sexes. Surprisingly, 77% of obese adolescents and 61% of overweight adolescents presented more than one cardiometabolic risk factor. Among the cardiometabolic risk factors, the ORs for elevated ALT levels were higher in obese boys and girls than in underweight/normal-weight adolescents. The prevalence of the clustering of cardiometabolic risk factors was significantly higher in overweight and obese boys than girls.

A study in the US showed that overweight/obese adolescents aged 12–19 years have an increased risk of high LDL-C, low HDL-C, high triglyceride levels, low HDL-C levels, high BP, and high glycated hemoglobin and FPG levels^5^. In that study, the proportion of cardiometabolic risk factors among overweight/obese adolescents aged 12–19 years was 4.7% for hypertension, 19.7% for high FPG levels, and 17.5% for high triglycerides. Another study of obese class III adolescents aged 12–17 years in the United States showed that prevalence of 7.7% for hypertension, 6.2% for type 2 diabetes, 9.0% for elevated ALT, 35.2% for dyslipidemia, and 13.6% for obstructive sleep apnea^23^. One study included 2327 obese European class III children and adolescents aged 8–19 years demonstrated that the proportion of hypertension, high FPG, and low HDL-C was 31.2%, 1.2%, and 65.0%, respectively^24^. In our study, the prevalence of cardiometabolic risk factors among overweight/obese Korean adolescents was 17.6% for hypertension, 15.8% for high FPG levels, 16.2% for elevated ALT levels, 23.3% for low HDL-C levels, and 26.9% for hypertriglyceridemia. These findings suggest that Asian adolescents tend to be more vulnerable to cardiometabolic risk factors at each BMI than Western adolescents, as seen in similar findings in adults^25–27^.

Odds ratios for obese adolescents with elevated ALT levels were prominent in both sexes. The risk of elevated ALT levels was four times higher in obese boys and approximately 13 times higher in obese girls than in underweight/normal weight adolescents. Approximately 28% of the obese boys and 14% of the obese girls had elevated ALT levels. Furthermore, this study showed that overweight and obese boys and girls conferred an increased risk of hyperuricemia; approximately 40% of obese boys had hyperuricemia. The latter is associated with obesity, high BP, insulin resistance, dyslipidemia, and chronic kidney disease^28,29^. To prevent NAFLD progression and hyperuricemia, early diagnosis and treatment of overweight and obesity in Korean adolescents are required.

Some studies, including those on adolescents in the United States, have shown sex differences in the risk ratio regarding the association between obesity and cardiometabolic risk factors^5^. Our study demonstrated that the risk ratios of cardiometabolic risk factors were higher in obese than in normal-weight boys, although the differences were not significant in girls. In addition, the study suggested that boys tend to develop cardiometabolic risk factors earlier than girls^5^. However, our study showed that both overweight/obese boys and girls had an increased risk ratio for cardiometabolic risk factors compared with adolescents with normal weight. This result suggests that Korean overweight/obese girls are not relatively less susceptible to obesity than overweight/obese boys. The prevalence of low HDL-C levels, elevated ALT levels, and hyperuricemia was much higher in boys than in girls.

Among overweight/obese children and adolescents in the United States, the proportion of those with ≥ 1, ≥ 2, ≥ 3, and ≥ 4 clustering cardiometabolic risk factors was 70%, 39%, 18%, and 5%, whereas this proportion was 51%, 19%, 5%, and 1%, among those with a BMI in the 85^th^–94^th^ percentile, respectively^15^. Nevertheless, our study showed that the prevalence of overweight/obese adolescents with ≥ 1, ≥ 2, and ≥ 3 clustering cardiometabolic risk factors was 70.1%, 40.4%, 17.8%, whereas this prevalence was 36.4%, 9.0%, and 2.0%, in the underweight/normal weight counterparts, respectively. These results indicate that the prevalence of clustering of cardiometabolic risk factors is much higher in Korean overweight/obese adolescents than in Korean underweight/normal-weight adolescents, despite the similar proportions of obese adolescents in the US. Moreover, this finding suggests that overweight/obese Korean adolescents tend to manifest and develop cardiometabolic risk factors earlier in life and seem to be at risk for progression. In addition, the prevalence of clusters 2, 3, and 4 in boys was approximately twice as high as that in girls. As overweight/obese Korean boys have a higher cardiometabolic risk, public-based management and treatment should be enhanced.

Several possible mechanisms are proposed to explain the clustering of cardiometabolic risk factors in obese adolescents. First, obesity increases visceral adiposity and lipolysis, leading to increased insulin resistance and glucose levels in the liver and muscle owing to high levels of free fatty acids and low levels of adiponectin^30^. Hepatic steatosis induces atherogenic dyslipidemia (high levels of triglycerides, small dense LDL-C, and low levels of HDL-C), is proinflammatory, and is associated with endothelial dysfunction and hypertension. In addition, increased lipolysis induces inflammation by increased tumor necrosis factor α (TNF α) and interleukin-6 (IL-6) levels and prothrombotic changes through plasminogen activator inhibitor-1 (PAI-1) secretion. Beta-cell failure and hypoinsulinemia in the pancreas are associated with the risk of type 2 diabetes and atherosclerosis^31^. These mechanisms explain the effect of obesity on clustered cardiometabolic risk factors in adolescents.

One study reported that people who remained obese from adolescence to adulthood had a two-to five-fold higher risk of type 2 diabetes, hypertension, and dyslipidemia than those who maintained a normal weight^32^. However, individuals who were obese or overweight in adolescence but had a normal weight in adulthood had similar risks of these diseases as those who maintained a normal weight from adolescence to adulthood^32^. These facts highlight the importance of weight management in overweight/obese adolescents and prevention of overweight/obesity in adolescence to reduce the risk of cardiovascular and metabolic diseases in adulthood.

The present study had some limitations. First, laboratory tests such as FPG, lipid profiles, and ALT levels were performed only once to define cardiometabolic risk factors, and we could not consider the daily fluctuations of these values. Second, because the health status and lifestyle of the participants were based on self-reported questionnaires, data may have included a recall bias. Third, although we might have included factors that influenced the study outcomes, not all confounding variables were considered.

In conclusion, Korean overweight/obese adolescents showed higher risks of not only having individual cardiometabolic risk factors, but also clustering of those risk factors. Our study suggests that Asian overweight/obese adolescents are more vulnerable to cardiometabolic risk factors than are Western overweight/obese adolescents. Cardiometabolic risk factors in adolescents can persist throughout adulthood and increase the risk of serious complications and mortality. Therefore, policies for the prevention and management of overweight/obesity in adolescents are urgently required.

## Data Availability

All data of this study are freely available at Korea Centers for Disease Control and Prevention. For data requests, please visit the following link for more information: https://knhanes.kdca.go.kr/knhanes/sub03/sub03_02_05.do
